# A case study of atypical Larsen syndrome with absent hallmark joint dislocations

**DOI:** 10.1002/mgg3.648

**Published:** 2019-03-27

**Authors:** Neslida Kodra, Callie Diamonstein, Natalie S. Hauser

**Affiliations:** ^1^ Inova Translational Medicine Institute, Inova Fairfax Hospital Virginia

**Keywords:** FLNB, joint dislocations, Larsen syndrome, phenotypic variability, skeletal dysplasia, whole‐exome sequencing

## Abstract

**Background:**

A family with skeletal and craniofacial anomalies is presented. Whole‐exome sequencing (WES) analysis indicated a diagnosis of Larsen syndrome, although their clinical presentation does not include the hallmark joint dislocations typically observed in Larsen syndrome.

**Methods:**

Patient consent for the sharing of de‐identified clinical and genetic information, along with use of photographs for publication, was obtained.

WES and variant segregation analysis by WES were performed by commercial laboratory, GeneDx (Gaithersburg, MD), on peripheral blood samples from the proband, her brother, and her parents using methods detailed on their website for test *XomeDx Whole Exome Sequencing Trio *(https://www.genedx.com/test-catalog/available-tests/xomedx-whole-exome-sequencing-trio/). WES uses next‐generation sequencing (NGS) technology to assess for variants within the coding regions, or exons, of approximately 23,000 genes. For the *FLNB* gene (NM_001457.3), 100% of the coding region was covered at a minimum of 10x. GeneDx uses Sanger sequencing to confirm NGS variants.

**Results:**

WES revealed a heterozygous pathogenic variant, p.Glu227Lys (c.679G>A), in the *FLNB* gene in three out of the four family members tested. This variant is associated with Larsen syndrome, a skeletal dysplasia condition with a wide range of phenotypic variability that usually includes congenital joint dislocations.

**Conclusion:**

This is a highly unusual presentation of Larsen syndrome in which the identifying hallmark trait is absent in the patients’ phenotypes.

## 1 INTRODUCTION

Larsen syndrome is a hereditary disorder that impacts the development of bones in the body. One in 100,000 babies is born with Larsen syndrome each year (Sajnani, Yiu, & King, 2013). Symptoms can vary, even within members of the same family; however, the condition is typically characterized by large‐joint dislocations and craniofacial anomalies. The hallmark feature of this condition is dislocations of the knee, hip, elbow, and wrist joints. Craniofacial abnormalities include hypertelorism, prominent forehead, depressed nasal bridge, and a flattened midface. Cleft palate, clubfoot, short stature, and spinal anomalies such as scoliosis and kyphosis are also very common.

Larsen syndrome is caused by mutations in the *FLNB* gene (OMIM 603381), which encodes the connective tissue protein, filamin B. This protein is thought to be involved in vertebral segmentation, joint formation, and endochondral ossification (Krakow, 2004). Five disorders have been described from pathogenic variants in the *FLNB* gene: spondylocarpotarsal syndrome (SCT), Larsen syndrome, type I atelosteogenesis (AO1), type III atelosteogenesis (AO3), and boomerang dysplasia (Robertson, 2008; Farrington‐Rock et al., 2006). However, as with many traditionally described gene‐disease associations, *FLNB*‐related disorders may represent an overall spectrum of disorders (Xu et al., 2018). In this spectrum of skeletal dysplasia conditions, Larsen syndrome is generally mildest, although within Larsen syndrome itself there is a range in phenotype.

While pathogenic variants in *FLNB* are typically inherited in an autosomal dominant fashion, autosomal recessive inheritance is also possible (Bicknell et al., 2006; Krakow et al., 2004; Robertson, 2008; Zhang et al., 2006). Autosomal recessive inheritance of *FLNB* variants is associated with SCT by causing a lack of expression in the *FLNB* protein (Robertson, 2008). The other four disorders, Larsen syndrome, AO1, AO3, and boomerang dysplasia, are associated with autosomal dominant or de novo variants in a gain‐of‐function manner (Farrington‐Rock et al., 2006). Heterozygous pathogenic variants in *FLNB* account for the majority of patients with Larsen syndrome; however, recently discovered homozygous pathogenic variants in *CHST3* (OMIM 603799) and *B4GALT7* (OMIM 604327) confirm the existence of recessive forms (Cartault et al., 2015; Hermanns et al., 2008). This case study will examine an atypical presentation of Larsen syndrome in which a family has a classic pathogenic *FLNB* variant with an autosomal dominant mode of inheritance, but is lacking the typical associated hallmark joint dislocations.

### 1.1 Case presentation

The proband, patient 1, was a 3‐day‐old female of mixed (Caucasian, African American, and Hispanic) ancestry born via Cesarean section at 35 weeks 5 days gestational age (GA) to a 26‐year‐old gravida 4 para 2 mother. The pregnancy was complicated by polyhydramnios starting at 21 weeks GA and continuing throughout the pregnancy. At 22 weeks 3 days GA, an intracardiac echogenic focus and nuchal thickening were noted. A subsequent ultrasound at 23 weeks 1 days GA showed the intracardiac echogenic focus and nuchal thickening had resolved but noted a left clubfoot. Another follow‐up ultrasound at 28 weeks 6 days was not consistent with left clubfoot.

At birth her APGAR scores were 8 and 8 at 1 and 5 min, respectively, but around 5 min, she developed respiratory distress requiring CPAP and admission to the NICU. She was found to have multiple anomalies and dysmorphic features, including cleft palate, flat midface, hypertelorism, creases under the eyes, a small nose with anteverted nares, arachnodactyly of fingers and toes, laterally deviated great toes, and mild pectus carinatum. She was in the 99th percentile for a length of 53.3 cm at birth. She failed her newborn hearing examination in both ears. She remained in the NICU for 6 weeks due to respiratory issues and slowing feeding.

A karyotype sent at the birth hospital was normal (46, XX). During her NICU stay, a head ultrasound performed 2 days after birth showed mild prominence of the lateral ventricles and no evidence of hemorrhage. A brain MRI performed at 5 weeks of age was overall normal. At 9 weeks of age she was evaluated for bilateral wrist contractures.

**Figure 1:**
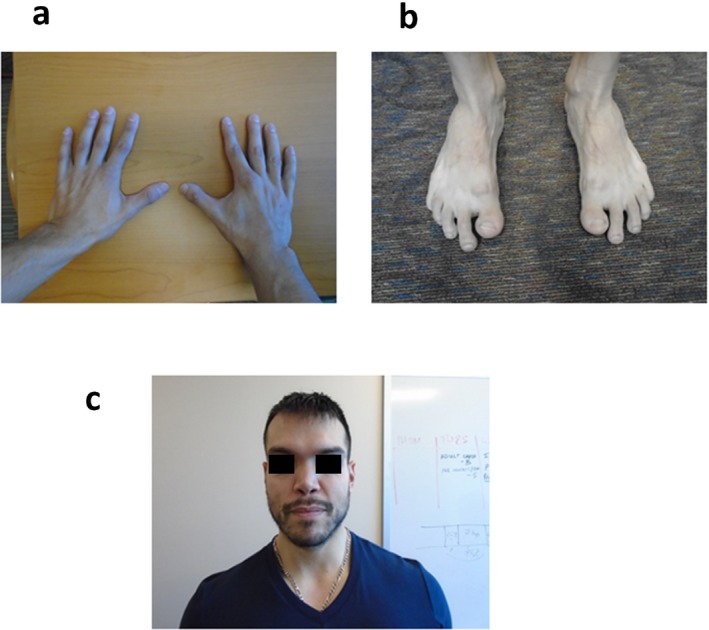
Notable physical features of patient 3. (a) Arachnodactyly observed in patient 3, noting long and slender fingers. (b) Bilateral laterally deviated great toes and arachnodactyly of toes on patient 3. (c) Dysmorphic facial features of patient 3. He has mild hypertelorism along with anteverted nares

**Figure 2:**
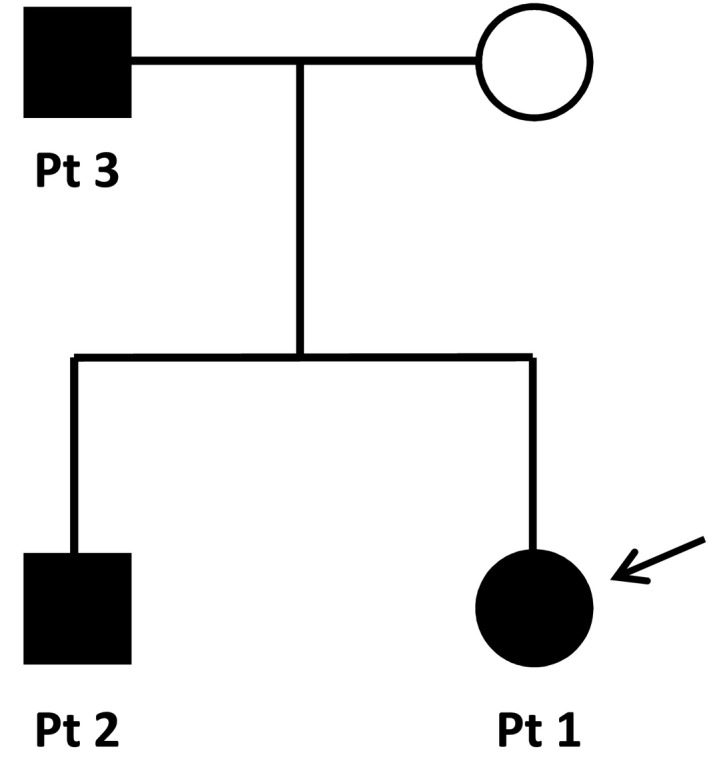
Pedigree of the affected family. Symptomatic individuals are in black. A pattern of autosomal dominant inheritance is visualized

Her family history is significant for a brother and father with similar physical features. Her 17‐month‐old brother, patient 2, previously presented at 1 day of life with a long neck, excess nuchal skin, large hands and feet with long fingers and toes, and pectus excavatum. He also had a flat midface and a saddle nose. A postnatal chromosome microarray analysis was normal. At the time, the father (patient 3) was found to have similar features including long fingers and toes, pectus excavatum, and laterally deviated great toes (Figure 1a,b). He also had anteverted nares and mild hypertelorism (Figure 1c). The father had been in generally good health his entire life. An autosomal dominant disorder was suspected due to the phenotypic similarities between the siblings and their father (Figure 2). Given the family history, we decided to offer further genetic testing to determine if there was an underlying hereditary syndrome responsible for their phenotypes.

### 1.2 Genetic analysis

A postnatal chromosome microarray analysis on patient 2 was normal. When patient 1 was born, whole‐exome sequencing (WES) was recommended, and the family agreed to a WES quad, including patient 1 (as proband), patient 2, the father (patient 3) and the mother. A heterozygous pathogenic variant, p.Glu227Lys (c.679G>A), was found in the *FLNB* gene in patient 1, patient 2, and patient 3 (Table 1). The mother was negative for this variant.

**Table 1:** Whole‐exome sequencing results for patients 1–3: autosomal dominant inheritance of a pathogenic variant in the *FLNB* gene

Patient	Gene	Variant	Coding DNA	Zygosity	Inherited from	Classification
1 (proband)	FLNB	p.Glu227Lys	c.679G>A	Heterozygous	Father	Pathogenic
2 (brother of proband)	FLNB	p.Glu227Lys	c.679G>A	Heterozygous	Father	Pathogenic
3 (father of proband)	FLNB	p.Glu227Lys	c.679G>A	Heterozygous	Unknown	Pathogenic

Note

The mother of the proband tested negative for this variant, indicating that patients 1 and 2 inherited the pathogenic variant from their father (patient 3).

The E227K variant in the *FLNB* gene has been reported in association with Larsen syndrome, as both a de novo variant and an inherited variant (Bicknell et al., 2006; Krakow et al., 2004; Zhang et al., 2006). This variant is located within a critical region, the actin‐binding region, where multiple other common pathogenic variants can be found (Daniel et al., 2012). Because this variant is a nonconservative amino acid substitution, it can change the secondary structure of the filamen B protein. Zhao, Shapiro, and Eto (2015) conducted functional studies that found that pathogenic variants in this region, including the E227K variant, can induce binding of the filamen B protein to F‐actin. This interaction causes F‐actin accumulation in the cell which can prevent normal skeletal development (Zhao et al., 2015). Therefore, the E227K variant meets the American College of Medical Genetics and Genomics criteria for pathogenicity.

## 2 DISCUSSION

This case highlights the variability in phenotypic expression of Larsen syndrome, even in regards to its hallmark feature of multiple joint dislocations. Dislocations of major joints are the most prominent clinical feature of Larsen syndrome and can be present soon after birth. Affected individuals may have several surgical procedures to stabilize the hip and knee joints. Later in adolescence, progressive degenerative arthritis of the joints can lead to painful contractures. Therefore, it is significant that none of the family members presented here had any dislocations, which is atypical of Larsen syndrome and many of the skeletal dysplasias similar to it.

Phenotypic variability in Larsen syndrome and similar skeletal dysplasias is very common. Tanteles, Dixit, and Dhar (2013) detailed a case of a *CHST3 *autosomal recessive Larsen syndrome where the proband was not born with any joint dislocations but went on to develop hip, elbow, and wrist arthritis during later childhood. Her maternal half‐brother on the other hand, was born with knee dislocations that resolved spontaneously (Tanteles et al., 2013). Indeed, much of the literature has focused on expanding the phenotypic range for this condition by detailing how unusual features may be present in a Larsen patient or typical features may be absent. Although uncommon, at least four nonrelated cases of dental issues have been reported in patients with this condition (Sajnani et al., 2013). Both conductive and sensorineural hearing loss are also uncommon features of Larsen syndrome, however, there have been a handful of reports of an association between the two (Herrmann, Kelly, Fied, & Strome, 1981; Nash, Majithia, Ujam, & Singh, 2012). Our proband had mixed hearing loss while both her father and brother had normal hearing. The proband also had skeletal and facial anomalies but had no dislocations or short stature. On the contrary, she was in the 99th percentile for height. Patient 3 had anomalies in the fingers and toes but he had no issues with walking. At 5 ft 9 in., he was also unaffected by short stature. Patient 2 had similar finger and toe anomalies as patients 1 and 3, but he was mildly delayed and used orthotics to correct his internal tibial torsion that was causing intoeing. While the family had some similar facial anomalies such as anteverted nares and hypertelorism, only the proband was born with cleft palate. This intrafamilial variability is quite common for this condition as family members with the same variant can have differing phenotypes, and it is a reflection of the overall phenotypic variability of Larsen syndrome.

Milder or unusual manifestations of the condition can result in misdiagnosis or no diagnosis at all. This particularly supports the use of broader genetic testing, such as WES, along with clinical evaluation to diagnose patients with atypical or variable clinical presentations. Recognition of this syndrome is important because of the implications for counseling. Affected individuals may require long term orthopedic care and monitoring. Therefore, in order to provide the best recommendations for care, it is important to accurately diagnose individuals with this condition. Expanding the range of signs and symptoms for this disorder is important to ensure that individuals who are missing certain features are still evaluated for the condition to ensure optimal management of their symptoms.

## CONFLICT OF INTEREST

The authors declare no conflict of interest.
